# Investigating pulmonary and non-infectious complications in common variable immunodeficiency disorders: a UK national multi-centre study

**DOI:** 10.3389/fimmu.2024.1451813

**Published:** 2024-09-10

**Authors:** Heba M. Bintalib, Sofia Grigoriadou, Smita Y. Patel, Leman Mutlu, Kavitha Sooriyakumar, Prashantha Vaitla, Elizabeth McDermott, Elizabeth Drewe, Cathal Steele, Manisha Ahuja, Tomaz Garcez, Mark Gompels, Alexandros Grammatikos, Archana Herwadkar, Rehana Ayub, Neil Halliday, Siobhan O. Burns, John R. Hurst, Sarah Goddard

**Affiliations:** ^1^ University College London (UCL) Respiratory, University College London, London, United Kingdom; ^2^ Department of Respiratory Care, King Saud bin Abdulaziz University for Health Sciences, Jeddah, Saudi Arabia; ^3^ King Abdullah International Medical Research Centre, Jeddah, Saudi Arabia; ^4^ Department of Immunology, Barts Health National Health Service (NHS) Trust, The Royal London Hospital, London, United Kingdom; ^5^ Clinical Immunology, Oxford University Hospitals National Health Service (NHS) Foundation Trust, Oxford, United Kingdom; ^6^ Clinical Immunology and Allergy, Department of Pathology, East Kent Hospitals University NHS Foundation Trust, Canterbury, United Kingdom; ^7^ Department of Allergy and Immunology, University Hospitals Birmingham National Health Service (NHS) Foundation Trust, Birmingham, United Kingdom; ^8^ Clinical Immunology and Allergy Department, Queens Medical Centre campus, Nottingham University Hospitals National Health Service (NHS) Trust, Nottingham, United Kingdom; ^9^ Regional Immunology Service of Northern Ireland, The Belfast Health and Social Care Trust, Belfast, United Kingdom; ^10^ Clinical Research Fellow, Newcastle University; Specialist Registrar Newcastle upon Tyne Hospitals NHSFT, Newcastle, United Kingdom; ^11^ Department of Immunology, Manchester University National Health Service (NHS) Foundation Trust, Manchester, United Kingdom; ^12^ The Bristol National Health Service (NHS) Immunology Allergy Centre, Southmead Hospital, North Bristol National Health Service (NHS) Trust, Bristol, United Kingdom; ^13^ Immunology Department, Division of Surgery and Tertiary Medicine, Salford Royal National Health Service (NHS) Foundation Trust, Salford, United Kingdom; ^14^ Clinical Immunology, Leeds Teaching Hospitals National Health Service (NHS) Trust, Leeds, United Kingdom; ^15^ University College London (UCL) Institute for Liver and Digestive Health, University College London, London, United Kingdom; ^16^ Sheila Sherlock Liver Centre, Royal Free London National Health Service (NHS) Foundation Trust, London, United Kingdom; ^17^ Department of Immunology, Royal Free London National Health Service (NHS) Foundation Trust, London, United Kingdom; ^18^ Institute of Immunity and Transplantation, University College London, London, United Kingdom; ^19^ Department of Immunology, University Hospitals North Midlands, Royal Stoke Hospital, Stoke-on-Trent, United Kingdom

**Keywords:** common variable immunodeficiency, interstitial lung disease, GLILD, bronchiectasis, noninfectious complications

## Abstract

**Background:**

Common Variable Immunodeficiency Disorders (CVID) encompass a spectrum of immunodeficiency characterised by recurrent infections and diverse non-infectious complications (NICs). This study aimed to describe the clinical features and variation in NICs in CVID with and without interstitial lung disease (ILD) from a large UK national registry population.

**Methods:**

Retrospective, cross-sectional data from a UK multicentre database (previously known as UKPIN), categorising patients into those with CVID-ILD and those with NICs related to CVID but without pulmonary involvement (CVID-EP; EP= extra-pulmonary involvement only).

**Results:**

129 patients were included. Chronic lung diseases, especially CVID-ILD, are prominent complications in complex CVID, occurring in 62% of the cohort. Bronchiectasis was common (64% of the cohort) and associated with greater pulmonary function impairment in patients with CVID-ILD compared to those without bronchiectasis. Lymphadenopathy and the absence of gastrointestinal diseases were significant predictors of ILD in complex CVID. Although the presence of liver disease did not differ significantly between the groups, nearly half of the CVID-ILD patients were found to have liver disease. Patients with CVID-ILD were more likely to receive immunosuppressive treatments such as rituximab and mycophenolate mofetil than the CVID-EP group, indicating greater need for treatment and risk of complications.

**Conclusion:**

This study highlights the significant burden of CVID-ILD within the CVID population with NICs only. The lungs emerged as the most frequently affected organ, with ILD and bronchiectasis both highly prevalent. These findings emphasise the necessity of a comprehensive and multidisciplinary approach in managing CVID patients, considering their susceptibility to various comorbidities and complications.

## Introduction

Common Variable Immunodeficiency Disorders (CVID) are an umbrella term for clinically defined immunodeficiencies characterised by low levels of serum immunoglobulins and heightened susceptibility to infections (1). CVID is the most common primary immunodeficiency in adults with a prevalence of 1:50,000 to 1:25,000 (2). Clinical presentations can be divided into CVID with infectious presentations only, and CVID with non-infectious complications (NICs) manifest by autoimmunity and/or immune dysregulation. Although immunoglobulin replacement therapy (IgRT) reduces both the number and severity of infections, it has limited influence on the immune dysregulation associated with CVID (3). NICs of CVID are complex and challenging to manage. They may arise prior to, or at diagnosis, or develop subsequently. Up to 60% of people with CVID experience NIC – depending on the definition of NIC used, but generally associated with increased risk of morbidity and mortality (4, 5). The challenge of NICs related to CVID is underscored by an overlapping spectrum of complications, which includes, progressive lung disease, lymphoproliferative disease, autoimmunity, granulomatous disease, and non-lymphoid malignancy (4–6).

Previous studies on CVID have laid a significant foundation for understanding the clinical picture of CVID and related NICs (5, 7–9). Notably, while the morbidity and mortality in CVID patients with infectious complications alone have improved with IgRT, patients with NIC have not benefitted from the treatment as they have similar survival rates to those observed in the 1970s (4). The role of lung diseases, such as CVID-related interstitial lung disease (CVID-ILD), has emerged as a significant concern, with several studies indicating a strong association between pulmonary complications and reduced survival in CVID patients (4, 10). Previous studies have reported the clinical presentation of CVID-ILD and assessed risk factors for developing ILD in all CVID population (8, 11, 12). However, CVID-ILD is considered the lung manifestation of systemic immune dysfunction, but not all people with systemic involvement get CVID-ILD. We wanted to compare people with systemic immune dysregulation with and without CVID-ILD.

Building on this established knowledge, our study aimed to describe the clinical features and variation in NICs in CVID with and without ILD from a large UK national registry population. Additionally, our focus extended to identifying the prevalence and risk factors for CVID-ILD among patients with NICs.

## Methods

This multicentre study was conducted in the UK and follows a retrospective, descriptive, cross-sectional design. Patients were identified in The United Kingdom Primary Immunodeficiency Network (UKPIN) registry. The inclusion criteria were a) adult patients (> 18 years) with confirmed CVID diagnosis based on ESID diagnostic criteria (available at http://esid.org/Working-Parties/Registry/Diagnosis-criteria), b) patients identified as 'granulomatous' in the UKPIN or as having multi-system disease associated with their CVID, and c) patients who provided consent for participation in the UKPIN registry. The exclusion criteria were no known NICs associated with CVID, and patients with bronchiectasis only – considered here as a complication of infection, rather than being a NIC.

Patients were divided into two groups for analysis:

A) CVID-ILD – CVID patients with evidence of ILD. Criteria were a clinical, radiological and/or pathological diagnosis of CVID-ILD according to the local clinicians. Typical radiological criteria include ground glass opacity, nodules, bronchial wall thickening, interlobular septal thickening and consolidation, and typical histopathological evidence comprising lymphoid interstitial pneumonitis, non-necrotizing granuloma, follicular bronchiolitis, lymphoid hyperplasia, and/or organising pneumonia.

B) CVID-EP – CVID patients with extrapulmonary NICs involvement only, such as autoimmune disorders, extrapulmonary granulomatous disease, gastrointestinal complications, or involvement of other organs.

### Data collection

A pre-filled spreadsheet containing the UKPIN registry number and date of birth was sent to the 25 relevant UK centres to obtain the necessary data from the patients’ records. For each patient, we collected demographic data and age at CVID diagnosis. Laboratory data, including levels of serum immunoglobulins (IgG, IgM, and IgA) at diagnosis, peripheral blood lymphocytes and B cell phenotyping were collected. We also collected organ-specific data related to the NICs. These included chronic lung diseases (CLDs), lymphoproliferative, gastrointestinal, liver, renal, skin, and neurological diseases. A history of autoimmunity, infection burden, treatments received, genetic data, malignancy and survival were also collected. The presence or absence of organ-specific disease was entered into the database based on the decision of the individual centre.

For CLDs, we asked if patients were diagnosed with CVID-ILD (according to the treating clinician) and/or bronchiectasis (with the presence of other NICs). The most recent lung function tests, including FEV1 (% predicted), FVC (% predicted), DLCO (% predicted), KCO (% predicted), and TLC (% predicted) were recorded and lung and extra-pulmonary biopsy results where available.

### Statistical analysis

Categorical variables were reported using count with percentage, with mean and standard deviation for continuous variables. The normality of distribution was tested using the Kolmogorov-Smirnov test. For the comparison between groups, an independent t-test was used to compare the means of continuous variables that were normally distributed (age, lung function results, immunoglobulin levels and lymphocyte counts) and the Mann-Whitney test was used where data were not normally distributed. Chi-square was used for categorical variables (gender, presence of extra-pulmonary involvement). Binary logistic regression was used to assess risk factors for CVID-ILD. Multivariable analysis was conducted using variables with p < 0.05 from the univariate analysis. SPSS version 28 was used for the analysis.

### Ethics

The study was conducted with approval from the UK Multicentre Research Ethics Committee (MREC), reference number 04/MRE07/68.

## Results

### Demographic data

We received data on 140 patients from 15 centres. We excluded 11 patients from the final analysis as their data showed bronchiectasis only (n = 6), no NICs related to CVID (n=2), and inappropriate data entry (n=3). 129 patients were therefore included in the analysis. Most of the cohort, 118 (91%), were white British.

Participants, all of whom had NICs, were stratified into two groups based on the presence or absence of CVID-ILD. 80 patients (62%) had CVID-ILD, and 49 patients (38%) had CVID with extra-pulmonary, non-infectious complications only (CVID-EP). Demographic characteristics are summarised in **Table 1**.

**Table 1:** Demographic and clinical data of groups.

	Cohort	CVID-ILD	CVID-EP only	P value
**n=**	129	80 (62)	49 (38)	
**Age/years**	56 ± 13	55 ± 14	58 ± 13	0.19
**Age at CVID diagnosis/years**	34 ± 14	39 ± 15	34 ± 14	0.08
Gender, N				0.22
**Male** **Female**	60 (47) 69 (53)	34 (43) 46 (57)	26 (53) 23 (47)	
**Genetic diagnosis, N**		11 (14)	4 (8)	
Lung				
Bronchiectasis, N	84 (65)	50 (62)	33 (69)	0.11
*Lung function tests*	80 (62)			
FEV1 (%predicted)	78 ± 23	81 ± 22	73 ± 24	0.09
FVC (%predicted)	87± 22	86 ± 22	88 ± 21	0.61
DLCO (%predicted)	61 ± 17	61 ± 17	63 ± 17	0.63
KCO (%predicted)	82 ± 17	83 ± 18	83 ± 16	0.81
TLC (%predicted)	85 ± 18	80 ± 18 (n = 21)	94 ± 15 ( n = 12 )	**0.03**
**Lung biopsy, N**	29	27 23 definitive	3 2 definitive	
Frequency of extrapulmonary involvement, N				
Splenomegaly	88 (68)	58 (72)	30 (61)	0.09
Splenectomy	15 (12)	9 (11)	6 (12)	0.86
Spleen size cm	18.8 ± 4.44	18.7 ± 4.31	18.9 ± 4.94	0.80
lymphadenopathy	76 (59)	55 (68)	21 (43)	**0.003**
Splenomegaly and lymphadenopathy	59 (46)	42 (53)	17 (35)	**0.04**
Liver	68 (53)	39 (48)	29 (59)	0.23
Gastrointestinal diseases	61 (47)	31 (39)	29 (59)	**0.03**
Renal	13 (10)	6 (7)	6 (12)	0.24
Skin disease	48 (37)	30 (37)	18 (37)	0.94
**History of autoimmunity**	75 (58)	52 (65)	22 (45)	**0.01**
Idiopathic Thrombocytopenic Purpura (ITP)	44 (34)	34 (42)	10 (20)	
Autoimmune Haemolytic Anaemia (AIHA)	19 (15)	18 (22)	1 (2)	
Neutropenia	13 (10)	8 (10)	5 (10)	
Other*	20 (15)	10 (12)	10 (20)	
Malignancy	24 (19)	10 (12)	14 (31)	0.01
Lymphoma	13	4	9	
skin	4	4		
Other	8	1 cecum 1 breast	2 gastric 2 vulval 1 pancreas 1 breast	
Deceased				
Causes of death	24 (19)	11 (14) 3 respiratory failure 2 liver failure 1 lymphoma 1 LMM 4 unknown causes	13 (27) 5 chest infection and resp failure 2 cancer 1 PML 1 liver failure 2 Multifactorial death 2 unknown causes	0.69

*Other autoimmunities include pancytopenia, microcytic anaemia, pernicious anaemia, CD4 lymphopenia, haemolytic anaemia, type 1 diabetes, inflammatory bowel disease, ulcerative colitis, sicca syndrome, and panhypopituitarism secondary to autoimmune hypophysitis and Evans syndrome. LMM, L. monocytogenes meningoencephalitis; and PML, progressive multifocal leukoencephalopathy.

Data are number (%) or mean ± SD.

Bold p-values indicate statistical significance (p < 0.05).

### Immunologic parameters and genetic diagnosis

The median serum immunoglobulin levels at diagnosis for the entire cohort were as follows: IgG, 1.35 g/L; IgA, 0.10 g/L; and IgM, 0.13 g/L. There was no significant difference between the groups (p > 0.05); detailed results are presented in **Table 2**. Total lymphocyte counts were within the normal ranges with no differences between groups. B cell phenotyping was reported in 53 (41%) patients. Among these, six exhibited very low B-cell numbers (<1%). In the EUROclass classification, borderline significant differences were observed between the groups (p = 0.05), with 26/36 (72%) CVID-ILD patients categorised as smB-Tr^norm^, indicating a reduction in switched memory and transitional B cells, compared to 7/17 (41%) in the CVID-EP group (13). The results for B cell phenotyping and EUROclass classification can be found in **Table 2**.

**Table 2:** Immunologic parameters at CVID diagnosis.

	Cohort	CVID-ILD	CVID-EP only	P value
Immunoglobulins*	N = 80	49	31	
IgG g/L	1.95 [UD - 3.07]	1.80 [UD - 3.05]	1.39 [UD - 3.30]	0.44
IgA g/L	0.10 [UD - 0.13]	0.10 [UD - 0.12]	0.09 [UD - 0.16]	0.16
IgM g/L	0.13 [UD - 0.31]	0.13 [UD - 0.31]	0.13 [UD - 0.40]	0.61
Peripheral blood lymphocytes	N = 106	N = 68	N = 38	
CD4 x 10^6^/ul	620 [358 - 1083]	631 [383 - 1050]	584 [267 – 1180]	0.58
CD8 x 10^6^/ul	296 [203 - 564]	283 [201 - 474]	318 [196 - 594]	0.59
CD19 x 10^6^/ul	119 [30 - 389]	126 [31 - 197]	97 [28 - 284]	0.81
B cell phenotyping	N = 38	N = 26	N = 12	
% of CD27- IgD+ (Naïve)	84.0 [73.0 - 91.0]H	81.0 [72.3 - 87.9]	86.8 [74.2 - 90.2]	0.45
% of CD27+ IgD+ (IgM Memory)	8.3 [4.8 - 15.4]	9.5 [5.4 - 16.2]	7.5 [4.9 - 16.2]	0.71
% of CD27+ IgD- (Sw. Memory)	1.9 [ 1.0 - 6.2]	2.0 [1.3 - 5.5]	1.9 [0.8 - 7.5]	0.80
Transitional B cells	2.5 [1.6 - 4.9]	2.4 [1.6 - 4.8]	1.9 [1.1 - 3.0]	0.61
CD21 low B cells	22.4 [ 15.3 - 42.9]	23 [14.6 - 49.9]	23.9 [16.1 - 34.3]	0.95
EUROclass classification, N		CVID-ILD	CVID-EP only	P value
EUROclass_A	N = 53	36	17	0.05
smB-Tr^norm^		26	7	
smB-Tr^hi^		6	2	
smB+		2	5	
B-		3	3	
EUROclass_B	N = 44	33	11	0.06
smB-21^lo^		28	6	
smB-21^norm^		3	2	
Smb + 21^lo^		1	3	
Smb + 21 norm		1	0	

*Normal immunoglobulins ranges for adults are IgG: 6 - 16 g/L, IgA: 0.9 - 2.8 g/L, IgM: 0.5 - 2.0 g/L.

Data are number (%) or median [IQR].

Seventy-eight (60%) of the patients underwent detailed genetic testing. Only 8 (13%) had a genetic diagnosis, of which 5 were identified using whole genome sequencing (WGS) and one using Whole exome sequencing (WES). Three patients had a defect in nuclear factor kappa B subunit 1 (NF-kB1). Two were found to have a transmembrane activator calcium-modulating cyclophilin ligand interactor (TACI) mutation. A mutation in interferon regulatory factor-2 binding protein 2 (IRF2BP2), inducible T-cell costimulatory (ICOS) deficiency, suppressor of cytokine signalling 1 (SOCS1), protein tyrosine phosphatase non-receptor type 2 (PTPN2) and cytotoxic T lymphocyte antigen 4 (CTLA4) were seen in one patient each.

### Chronic lung diseases

The lungs were the most frequently affected organ, impacting 113 (88%) of our cohort. Bronchiectasis was commonly observed across the entire cohort, affecting 84 (64%). Among the CVID-ILD and CVID-EP groups, the prevalence of bronchiectasis was 62% and 69%, respectively. CVID-ILD was noted in 80 patients (62%) of the cohort.

**Figure 1:**
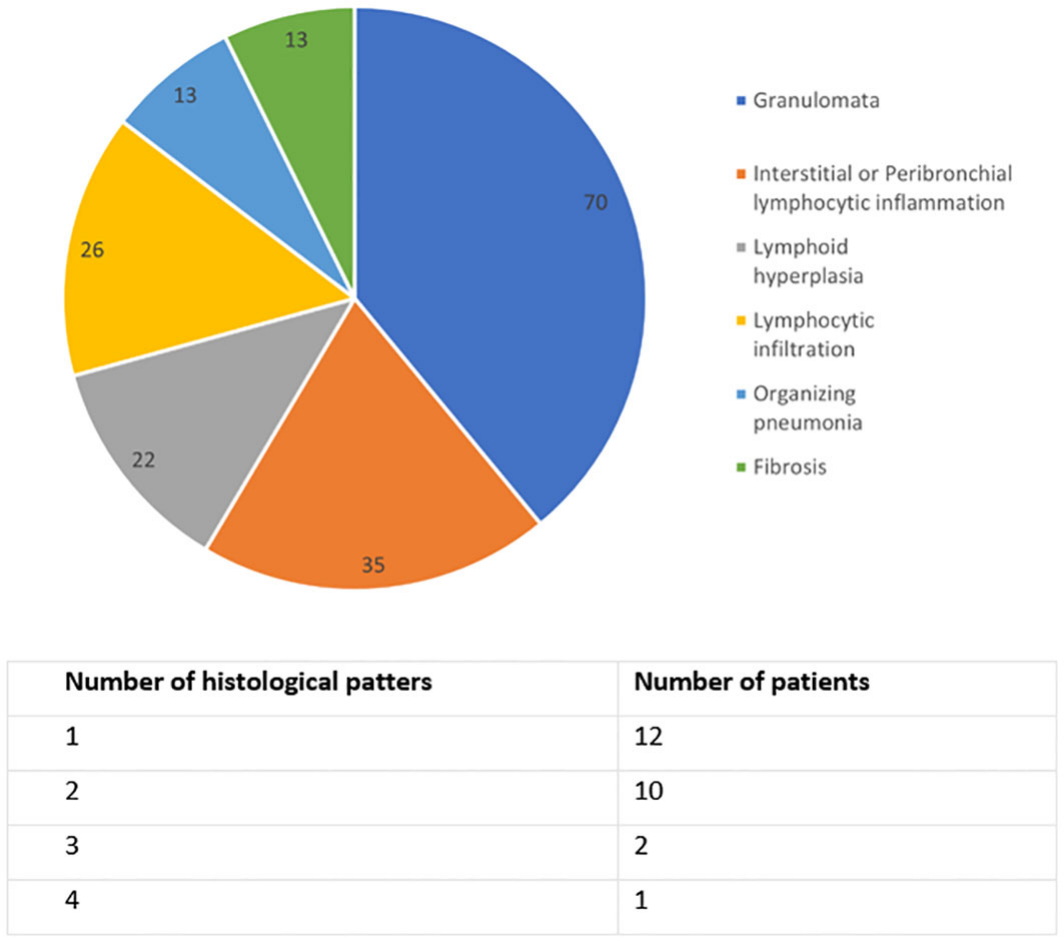
Histological lung biopsy results of CVID-ILD group, n=23. Data are in percentage, and the sum exceeds 100% because some patients had multiple findings.

Lung biopsy had been performed in 27 (34%) patients with CVID-ILD. A diverse range of histological patterns were identified. 17 (74%) exhibited pulmonary lymphoid hyperplasia, which included features such as interstitial or peribronchial lymphocytic inflammation, lymphocytic infiltration, and lymphoid hyperplasia, either singly or in combination. 16 (70%) patients displayed granulomata. Lung biopsy results are summarised in **Figure 1**.

Differences in lung function between the CVID-ILD and CVID-EP groups are summarised in **Table 3**. The two groups did have a statistically significant difference in TLC: CVID-ILD have significantly lower TLC % than the CVID-EP, but both were within normal limits (mean ± SD = 80 ± 18% and 94 ± 15%, respectively, p=0.03).

**Table 3:** Comparison of the CVID-ILD and CVID-EP groups with and without bronchiectasis.

	CVID-ILD		P value*	CVID-EP only		P value**
	CVID-ILD only	+Bronchiectasis		Extrapulmonary only	+Bronchiectasis	
**n=**	31	49		15	34	
Age /years	50 ± 14.92	58 ± 14.45	0.19	53 ± 12.94	60 ± 12.12	0.07
Age at CVID diagnosis/years	40 ± 15.02	38 ± 14.45	0.38	34 ± 9.52	35 ± 14.73	0.82
Gender			0.021			0.98
Male Female	18 (58) 13 (42)	16 (32) 34 (68)		8 (53) 7 (47)	18 (53) 16 (47)	
Lung function tests						
FEV1 (%predicted)	88 ± 23	76 ± 20	**0.03**	96 ± 11	62 ± 20	**< 0.001**
FVC (%predicted)	93 ± 22	81 ± 21	**0.02**	108 ± 12	78 ± 18	**<0.001**
DLCO (%predicted)	68 ± 15 N=30	56 ± 18 N=40	**0.00**	69 ± 17 N=9	60 ± 17 N=20	0.17
KCO (%predicted)	89 ± 15	77 ± 19	**0.005**	81 ± 15 N=10	84 ± 17 N=19	0.69
TLC (%predicted)	81 ± 19 N=10	80 ± 17 N=11	0.87	99 ± 16 N=4	91 ± 14 N= 8	0.40
Deceased	2 (6)	9 (18)	0.08	2 (13)	11 (32)	0.18

*CVID-ILD only vs. CVID-ILD + bronchiectasis. **Extrapulmonary only vs. with bronchiectasis.

Data are reported as mean (SD) or number (%).

*Bold p-values indicate statistical significance (p < 0.05).

The CVID-ILD group who also had bronchiectasis exhibited had significantly lower FEV1%, FVC%, DLCO%, and KCO% values (mean ± SD = 76 ± 20%, 81 ± 21%, 56 ± 18%, and 77 ± 19%, all p <0.05, respectively) compared to those without. Furthermore, the CVID-EP group with bronchiectasis also demonstrated significantly lower FEV1% and FVC% than CVID-EP without bronchiectasis (mean 62 ± 20% and 78 ± 18% vs. 96 ± 11% and 108 ± 12%, p < 0.001).

### Multi-organ involvement

The prevalence of NICs by group is summarised in **Table 1**. Splenomegaly was the most common complication, seen in 88 patients (68%). Splenectomy had been performed in 9 (11%) and 6 (12%) of the groups. Lymphadenopathy (at any site) was seen in 76/129 (59%) of the cohort. It was significantly more common in CVID-ILD (55 (68%) vs. 21 (43%), p = 0.003). Additionally, the occurrence of both splenomegaly and lymphadenopathy was significantly more frequent in CVID-ILD than in CVID-EP (42 (53%) vs. 17 (35%), p = 0.04).

Autoimmune manifestations were found in 75 (58%) of the cohort. A history of autoimmunity was significantly more common in CVID-ILD than in CVID-EP (52 (65%) vs. 22 (45%), p = 0.01). Among the CVID-ILD, ITP (42%) and AIHA (22%) were the most common manifestations.

Hepatic involvement such as persistently elevated liver enzymes, portal hypertension and/or abnormal imaging were present in 68 (53%) of the whole cohort. Hepatomegaly was present in eight patients and radiological portal hypertension in nine. Liver biopsy had been performed in 39 patients (57% of those with hepatic disease), revealing nodular regenerative hyperplasia (NRH) in 19 patients (48% of those who had liver biopsy), granulomas in 15 (3 patients with granuloma also had NRH), and fibrosis in 11. Out of 20 CVID-ILD patients who had a liver biopsy, 5 had histologically confirmed granulomatous disease, 5 had fibrosis, and 8 had NRH.

Gastrointestinal diseases (GI) (as defined by the treating clinician) were observed in 61 (47%) of the cohort and were significantly more common in the CVID-EP than the CVID-ILD group (29 (59%) vs 31 (39%), p = 0.03). Endoscopic examinations of both upper and lower tracts were performed in 38 patients, revealing that 29 (76%) experienced pathology affecting the small or large intestines. Small intestine diseases were noted in 18 patients (14% of the entire cohort), with the most prevalent histological findings being villous atrophy in 10 patients, nodular lymphoid hyperplasia in five, and intraepithelial lymphocytosis and non-specific inflammation in three patients each. Large intestine disorders predominantly comprised non-specific inflammation in five patients, and granulomas and nodular lymphoid hyperplasia in two patients each. GI plasma cells were absent in six patients; three were in the CVID-ILD group. Chronic norovirus infections were reported in four patients, of which three patients were CVID-ILD, and cryptitis was found in one patient with CVID-ILD. Features consistent with coeliac-like disease were found in 13 patients (10% of the entire cohort) and six were in the CVID-ILD group, with villous atrophy evident in only 5 patients. Histological features typical of Crohn's disease were identified in two patients, one in each group. In the CVID-ILD group, biopsy-proven granulomas were found in two patients.

Skin conditions were reported in 48 (37%) of the cohort, with biopsies performed on 17 patients. Lichen planus was diagnosed in three patients, granulomatous inflammation in eight, and nodular prurigo in two. Autoimmune complications such as alopecia and vitiligo were present in three and five patients with skin diseases respectively. Neoplastic skin diseases were found in three patients; one had malignant melanoma, another had squamous cell carcinoma of the ear, and one had carcinoma of scalp.

Renal disorders were detected in 13 (10%) of the cohort. Biopsy results were accessible for three patients, revealing interstitial nephritis and granulomatous interstitial nephritis in two patients each and non-caseating granulomas in one patient. Five patients (2%) had neuromuscular conditions. Inflammatory myopathy, cerebellar granuloma, and myasthenia gravis were reported in one patient each. The patient with cerebellar granuloma was also found to have central nervous system (CNS) lymphoma. Two patients had transverse myelitis.

Malignancy was found in 24 (19%) patients of the cohort (**Table 1**) and was significantly more common in the CVID-EP group (14 (31%) vs 10 (12%), p = 0.01).

Granulomas were most frequently observed in lung tissue (n = 16), followed by the liver (n = 14), lymph nodes (n = 10), and skin (n = 8). Less commonly, granulomas were found in the brain (n = 1) and eye (n = 1).

24 (19%) of the patients had died. The breakdown of known causes of mortality included respiratory failure in nine patients, cancer and lymphoma in three patients, liver failure in two patients, multiorgan involvement in four patients, and progressive multifocal leukoencephalopathy in two patients.

24 (19%) of the patients had died. The breakdown of known causes of mortality are summarised in **Table 1**.

Regarding treatment strategies, the majority received IgG replacement therapy across all groups, with rates of 100% in CVID-ILD and 92% in CVID-EP. The use of systemic steroids, azathioprine, and TNF-a mAb was higher in CVID-ILD. Rituximab and Mycophenolate Mofetil were used significantly more in CVID-ILD, reflecting the increased use of immunosuppressants among patients with ILD (**Table 4**).

**Table 4:** Distribution of treatment preferences among cohort groups.

	Cohort	CVID-ILD	CVID-EP only	P value
n=	129	80 (62)	49 (38)	
Treatment n (%)				
**IgG replacement**	119 (92)	80 (100)	45 (92)	
**Systemic steroid/total**	84 (65)	57 (72)	27 (55)	0.11
Current		26 (32)	8 (16)	
Previously		31 (39)	19 (39)	
**Rituximab/total**	32 (25)	26 (32)		0.02
Current		4 (5)		
Previously		22 (27)	6 (12)	
**Azathioprine/total**	24 (19)	18 (23)		0.16
Current		3 (3)		
Previously		15 (19)	6 (12)	
**Mycophenolate mofetil/total**	27 (21)	22 (28)	5 (10)	0.02
Current		15 (19)	2 (4)	
Previously		7 (9)	3 (6)	
**TNF-a mAb/total**	4 (3)		4 (8)	0.22
Current			1 (2)	
Previously		2 (2)	3 (6)	
**prophylactic antibiotic (current)**	118 (91)	73 (90)	45 (98)	0.33

Data are number (%).

Bold p-values indicate statistical significance (p < 0.05).

### Univariate and multivariate analysis

Univariable analysis results are summarised in **Table 5**. In a multivariable analysis adjusted for age and gender, the presence of lymphadenopathy remained significantly associated with increased odds of ILD (OR 2.59, 95% CI: 1.01-6.63, p = 0.04). Conversely, patients with histological evidence of CVID-associated GI disease were significantly less likely to have ILD, with an odds ratio of 0.30 (95% CI: 0.12-0.74, p = 0.01).

**Table 5:** Association of non-infectious complications of CVID with ILD.

	OR (95% CI)	P value
Lymphadenopathy	3.27 (1.52-7.00)	0.002*
Splenomegaly and lymphadenopathy	2.12 (1.01-4.43)	0.04
History of autoimmunity	2.70 (1.22-5.98)	0.01
Gastrointestinal diseases	0.44 (0.21 – 0.91)	0.03*

(*) variables in multivariable analysis with p < 0.05. Lymphadenopathy OR (95% CI) = 2.59 (1.01 6.63), and gastrointestinal diseases = 0.30 (0.12 – 0.74).

## Discussion

The study presents a comprehensive analysis of a UK multicentre cohort of 129 patients and explores the characteristics and prevalence of non-infectious complications related to CVID, especially in the context of interstitial lung disease. Our results can be summarised as follows: 1) chronic lung diseases were the predominant complications in our CVID cohort with NICs, 2) the presence of co-existent bronchiectasis in CVID-ILD was associated with the most abnormal lung function, 3) lymphadenopathy increased the risk of CVID-ILD, supporting previous reports (8), and 4) GI complications were significantly less common in the CVID-ILD group.

B cell phenotyping is an essential component of comprehensive evaluation in CVID. Studies have shown that CVID patients with reduced switched memory B cells face a higher risk of developing non-infectious complications such as splenomegaly, granulomas, and autoimmunity (13, 14). Additionally, expansion of CD21^low^ B cells was also associated with splenomegaly, autoimmunity and CVID-ILD (11–13, 15). Our findings demonstrated a borderline significant difference between CVID-ILD and CVID-EP patients, with more CVID-ILD patients displaying reduced levels of switched memory B cells, considering that the CVID-EP group had NICs. Furthermore, although no significant differences were found in the percentage of CD21low B cells, expansion was notably higher in the CVID-ILD group. These results align with Hartono et al., who reported a significantly higher percentage of CD21low B cells in patients with GLILD compared to controls in their study evaluating the correlation between GLILD and various clinical and laboratory parameters. They also identified an increase in the percentage of CD21low B cells as a predictor of GLILD development, along with low IgA levels, the presence of splenomegaly, and either ITP or AIHA. Cinetto et al. reported similar findings and their productive models added DLCO% predicted to predicted GLILD. These findings highlight the importance of detailed immunological profiling to identify potential risk factors for serious complications in CVID patients, facilitating targeted monitoring and intervention strategies.

The prevalence of NICs has previously been reported in more than 60% of people with CVID (5, 9, 16), depending on the definition used. In a general CVID cohort, the overall prevalence of splenomegaly ranges from 13% to 47%, lymphadenopathy from 27% to 35%, and immune cytopenia from 10 - 33% (5, 7, 9, 16). Among our CVID cohort with NICs only, splenomegaly, lymphadenopathy, and immune cytopenia were observed in 68%, 59%, and 58%. Similar to previous reports, ITP and AIHA were the most commonly reported autoimmune cytopenia (4–6, 9).

Chronic lung diseases were the most common complication affecting patients in our cohort, reported in 88%. Previous studies have indicated a prevalence of CLDs, including CVID-ILD and bronchiectasis, in all CVID of 28 – 60% (4, 5, 7, 16). CVID-ILD was found in 62% of our cohort, significantly higher than previous studies that reported the prevalence in the context of all CVID 8-20% (4, 5, 7, 10, 15–17). This higher prevalence in our cohort may be attributed to our population selection and, therefore, this figure cannot be generalised to reflect prevalence in other CVID populations. Almost all of the CVID-ILD group also had extrapulmonary immune involvement. This adds to the evidence suggesting that ILD in CVID is linked to a systemic immune dysfunction encompassing various extrapulmonary manifestations and immune dysregulation (8, 11, 12, 18). Despite the high prevalence of CVID-ILD, only one-third of the patients had this confirmed with lung biopsy and the need for lung biopsy in patients suspected of having CVID-ILD remains debated.

A comparison of lung function between the CVID-ILD and CVID-EP groups did not show significant differences. We note that DLCO was below the normal range even in the CVID-EP group without known bronchiectasis, suggesting that some of this group may have undiagnosed lung disease. Our data support recommendations of establishing baseline chest CT and lung function tests at the diagnosis of CVID (1). This baseline will enable future comparisons, allowing early detection and timely interventions to prevent irreversible lung damage (7, 19). Moreover, our data supports the necessity of continuous monitoring even for those with no respiratory symptoms.

Bronchiectasis was reported in 64% (n = 84) of our cohort, consistent with the prevalence in unselected CVID, ranging from 11-90% (7, 16, 20, 21). Bronchiectasis results from progressive damage to the bronchial wall caused by recurrent lower respiratory infections, occurring before initiation of, and sometimes progressing despite adequate IgRT (3). It has been reported as a significant contributor to increased morbidity and mortality in CVID (20, 21). Sperlich et al. reported that the presence of bronchiectasis in CVID only patients was significantly correlated with reduced lung function measured using spirometry, and diffusion capacity, consistent with our finding (20). To the best of our knowledge, no previous study has evaluated lung function tests in CVID-ILD patients with and without bronchiectasis. Our analysis revealed a significant reduction in spirometry and gas transfer in CVID-ILD with bronchiectasis compared to those without. This indicates a more profound impact on lung function in this group which emphasizes the importance of initiating treatment or preventive measures when bronchiectasis is present to mitigate further lung function decline.

Unfortunately, our analysis did not distinguish between types of bronchiectasis. For instance, ‘traction’ bronchiectasis (a feature of ILD) could account for the greater impairment in lung function, and this is associated with reduced survival in ILD (22, 23). Additional research is warranted to determine the effect of bronchiectasis on CVID-ILD. Part of the challenge here is the terminology of non-infectious and infectious complications in CVID. Although usually a consequence of infection, ‘free standing’ bronchiectasis has been considered a non-infectious complication with ‘infectious complications’ usually reserved for acute infection events.

Although splenomegaly was a commonly reported complication affecting 68% of our CVID cohort, it was not associated with ILD, contrary to previous reports that indicated a significant association (8, 10–12). This discrepancy is likely due to the absence of a comparison group without NICs. However, we did note that the prevalence of combined splenomegaly and lymphadenopathy was significantly higher in CVID-ILD. The prevalence of splenomegaly in CVID-ILD was 72%, consistent with previous reports at 58-69% (8, 11, 12).

Despite limitations, both lymphadenopathy and a history of autoimmunity were independently associated with CVID-ILD in the univariate analysis. However, when adjusting for cofounders, only lymphadenopathy retained statistical significance and was consistent with previous studies. This implies that CVID patients with lymphadenopathy are at an increased risk for developing ILD, supporting the need for careful monitoring and maintaining a high level of suspicion. This may also suggest linked disease mechanisms and systemic involvement. However, It is also possible that clinicians look more carefully for lung involvement in people with other NICs.

Organ-specific involvement was prominent in our cohort. The presence of liver disease in 52% of the patients and gastrointestinal diseases in 47% highlights the systemic nature of this CVID cohort, with skin, renal, and neurological disorders less frequently seen. The most common histological findings on liver biopsy were NRH and granulomas, consistent with recent reports (5, 24–26). A recent study by Halliday et al., which reported the clinical features of 91 patients with CVID-related liver diseases, reported a significant association between liver disease and the presence of ILD (26). Specifically, patients with ILD had 2.82 times higher odds of developing liver disease compared to those without this comorbidity. Although not a statistically significant comparison in our cohort, nearly half of our CVID-ILD patients were reported to have liver involvement. This highlights the importance of careful monitoring and management of liver health in patients diagnosed with CVID-ILD.

Interestingly, biopsy proven GI diseases were found to be less common in patients with CVID-ILD. There is no previous similar report. Khan et al. reported that autoimmune cytopenias and chronic lung disease were more common in patients without GI symptoms, though they did not specify which chronic lung diseases were involved (27). Several factors may contribute to our findings. The heterogeneity of CVID could play a role, as these associations might arise from differences in immune system dysfunction between patients. Furthermore, the CVID-ILD group received more immunosuppressant therapy compared to the CVID-EP group, which may indicate differences in disease severity or presentation between the groups. Thus, our findings may be influenced more by the use of treatments than by the natural progression of the disease, as treatments might reduce or mask GI symptoms. This makes it challenging to draw definitive conclusions about this finding, and further larger studies are needed to confirm and explain this association.

Neoplastic diseases were observed in 19% of our CVID cohort. The pooled prevalence of neoplastic diseases in overall CVID is said to be around 10%, with non-Hodgkins lymphoma most common (1, 2, 5, 12, 25). Recently, Burnes conducted a study on the frequency of cancer in CVID. They found that immune dysregulation, such as ILD, atrophic gastritis, and arthritis, were risk factors for malignancy in CVID (28). This emphasises the importance of being alert to cancer in this population.

Our study revealed notable differences in the use of treatments. The CVID-ILD group showed a higher tendency to be treated with immunosuppressant therapies. The significant use of rituximab and mycophenolate in CVID-ILD highlights the preference for interventions to manage the immune dysregulation and inflammation associated with CVID-ILD. This approach is consistent with established research supporting immunosuppression for ILD (29–31). In contrast, similar evidence for treatment in other complications such as liver disease is less well developed.

Our study, while comprehensive, has limitations that warrant consideration. Our cohort consisted predominantly of white British individuals (91%). This demographic skew reflects the population distribution within the UK and may limit the generalizability of our findings to more ethnically diverse populations. The potential impact on our results includes the possibility that genetic or environmental factors specific to this demographic could influence the prevalence and manifestation of CVID and associated complications. Moreover, The accuracy and completeness of data gathered in retrospect can be incomplete. We acknowledge the possibility of variation in the data collection due to the many specialists involved from 15 different centres. Some details, such as lung radiological data and observation times, were either unreported or inconsistently reported, potentially affecting the reliability of our findings. This includes the gap between diagnosis and the start of IgRT, as well as diagnostic delays. Information including the reasons of using immunosuppressants and the effect of treatments were difficult to interpret. We were not able to comment of the sequence of complications in our cohort.

In conclusion, the study contributes to understanding NICs in CVID, shedding light on the complex nature of this condition. The lungs emerged as the most frequently affected organ, with ILD and bronchiectasis both highly prevalent. Notably, the co-occurrence of bronchiectasis and ILD was linked to greater impairment in lung function. Lymphadenopathy and the absence of gastrointestinal diseases were associated with a higher risk of CVID-related ILD. These findings emphasise the necessity of a comprehensive and multidisciplinary approach in managing CVID patients, considering their susceptibility to various comorbidities and complications.

## Data Availability

The raw data supporting the conclusions of this article will be made available by the authors, without undue reservation.
